# Impact of retrofitting work on vulnerability reduction of local buildings in Kabul, Afghanistan

**DOI:** 10.4102/jamba.v13i1.1062

**Published:** 2021-06-24

**Authors:** Mohsen Mohammadi, Toshio Fujimi

**Affiliations:** 1 Department of Advanced Industrial Science, Graduate School of Science and Technology, Kumamoto University, Kumamoto, Japan

**Keywords:** retrofitting, non-engineered buildings, training, local masons, Kabul, damage ratio, behaviour modifier factor, vulnerability index

## Abstract

Rapid urbanisation of Afghan cities without proper construction regulation has exposed their population to a high risk of damage from disasters such as earthquakes. With the growing construction of local non-engineered buildings and an existing level of hazard of 0.8 g, a high risk of casualties and building damage threatens Kabul in the event of a disaster. This study reports and evaluates a recent retrofitting project in Kabul City by ‘Project for City Resilience’, carried out under the supervision of the United Nation Human Settlements Program (UN-Habitat) for 48 retrofitted sun-dried clay brick masonry buildings in Kabul. The project was executed by local masons and welders who were trained as a part of the project, and the main tasks included installation of an additional steel frame, additional reinforced concrete foundation ring, ceiling replacement and wall strengthening (via mesh and plaster). After a visual assessment of retrofitted buildings considering the original retrofitting design and actual work done, a vulnerability index for retrofitted buildings was developed based on a behaviour modifier factor, which was assigned to each retrofitting activity using a combination of values and a proportion of scores for each retrofitting activity. The results indicate that training of local masons and welders to undertake retrofitting activities could decrease the damage ratio by 15% – 20% for peak ground acceleration values of 0.3 g and higher. The methods mentioned in this study can be used to make existing sun-dried clay brick masonry buildings sufficiently resistant to earthquakes of moderate-to-severe intensity.

## Introduction

**FIGURE 1:**
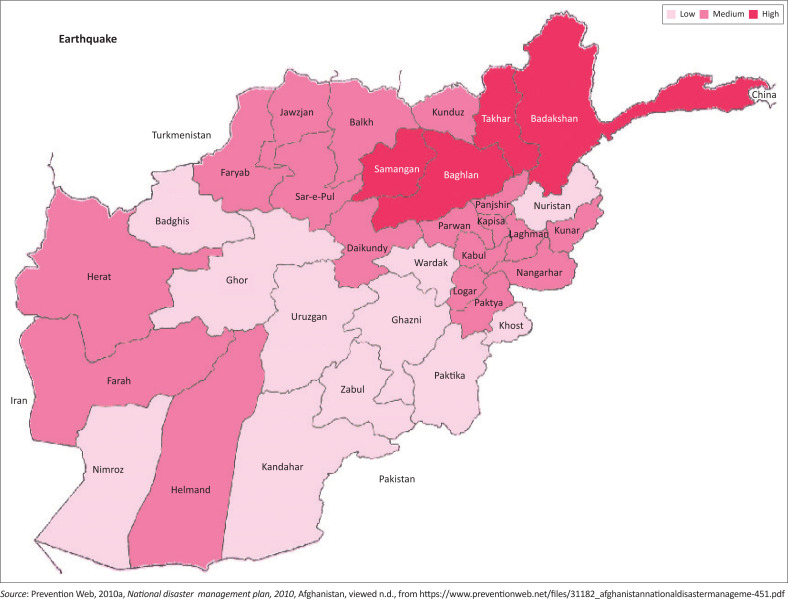
Earthquake zoning map of Afghanistan.

**FIGURE 2:**
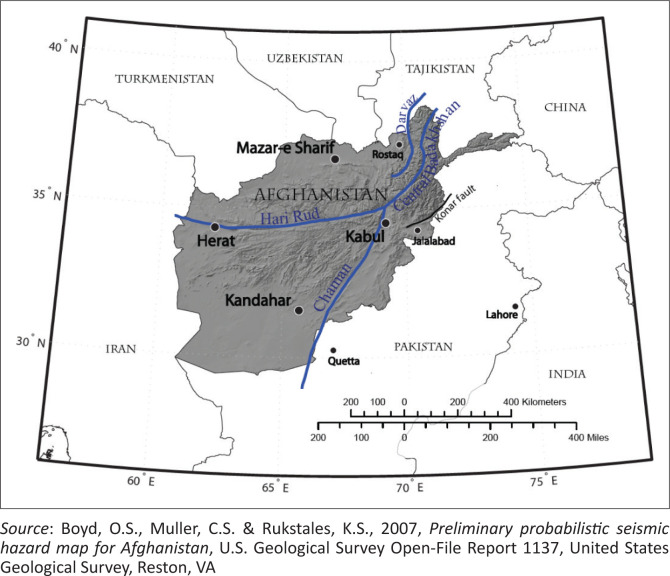
Map of Afghanistan with seismic faults.

Afghanistan is a land-locked and mountainous country located between Central Asia and South Asia. From 1979 to 2001, this country has experienced civil war. However, currently, different types of natural hazards such as floods, earthquakes, intense heat and drought are threatening Afghan cities. These natural calamities are a result of the concentration of population, industry and infrastructure combined with inadequate disaster risk reduction countermeasures. Being located on two major active faults with high potential of experiencing devastating earthquakes, the entire country, especially the east and northeast regions, is predicted to experience devastating earthquakes that may lead to extensive loss of life and property. Because of the lack of proper construction standards and poor enforcement of existing rules, many buildings cannot even withstand a moderate earthquake (Ministry of Urban Development Affairs 2015). Records of natural hazards in Afghanistan from 1954 to 2006 indicate that 112 extensive hazards have led to 22 000 fatalities and around 11 million people have been affected in the country (Prevention Web 2010a and b). According to the seismic zonation of the country, shown in Figure 1, the eastern and north-eastern areas of the country are identified to be located in a region of high seismicity; this includes Kabul City, which has undergone rapid urbanisation with the construction of unsafe local structures in recent years (Prevention Web 2010a). A field investigation and technical analysis of the current main active faults of Afghanistan, shown in Figure 2, together with the use of a source model catalogue of earthquakes that occurred in the past were conducted in 2007 by the United States Geological Survey (USGS). The analysis was conducted using the same approach used to create a hazard map for the United States. The outcome of the report presented a seismic hazard map and hazard curve for Afghanistan. It shows the extent and level of earthquake hazard across the country at different return periods using peak ground acceleration (PGA), which represents the maximum ground acceleration during an earthquake and is an important parameter to be considered for earthquake-resistant building design. The report declared that an estimated 0.50 g PGA, equivalent to an intensity VIII earthquake with 2% probability of exceedance in 50 years, can devastate many brick masonry dwellings and lead to extensive property damage and human loss in Kabul City (Boyd, Muller & Rukstales 2007). In another seismic hazard assessment conducted by the International Institute of Earthquake Engineering and Seismology of Iran (IIEES) at the city level for Kabul, the level of seismic hazard for the city was indicated to be higher than that reported in previous studies. In Kabul, it is expected that the PGA would be 0.8 g with an earthquake of magnitude 7.5 in a situation where construction practice in Afghanistan is still below acceptable norms (Ashtiany et al. 2019). An evaluation of seismic risk in a part of Kabul city considering the current local dwelling construction reflects the high risk associated with adobe and masonry houses (Mohammadi & Fujimi 2016).

Based on the above discussion, it is evident that development and enforcement of appropriate codes and bylaws for building construction as well retrofitting of current vulnerable structures would help in significantly reducing the vulnerability of Afghan communities to disasters. This issue was reflected as part of activities for disaster risk reduction by the Project for City Resilience (PCR), which was conducted by the United Nations Human Settlements Program (UN-Habitat) from April 2017 to March 2019 in the two major cities of Kabul and Mazar-i-sharif in cooperation with the Government of Afghanistan, with the objective of assisting the National Unity Government in making Afghan cities safe, sustainable and resilient to natural hazards. The author was involved in the structural activities of the project as a disaster risk reduction specialist during this period. As a part of such activities, 48 non-engineered masonry houses in Kabul City and another 50 houses in Mazar-i-sharif were selected via a modality of selection in cooperation with local authorities and subsequently retrofitted. The retrofitting activities mainly included an additional steel frame, an additional reinforced cement concrete foundation ring, ceiling replacement and wall strengthening (via mesh and plaster). The process of preparing design drawings for retrofitting was supported by UN-Habitat’s Iran office. The equipment and construction materials were provided by the project and implemented on ground by local masons and welders who were also trained by the project. One of the important goals of the project was to enhance the capacity of local communities and local governments to become familiar with the deficiencies in local construction and to reduce seismic risk (UN-Habitat & PCR 2019).

This article gives a brief profile of Kabul City, describes the existing level of seismic hazard and provides an overview of some of the existing non-engineered housing typologies and construction materials that exist in the areas targeted by the PCR. Then, the details of the selection process and retrofitting activities applied to the 48 houses in Kabul will be discussed. The article then reports how actual retrofitting activities conducted on-site were compared with the original retrofitting design, and a behaviour modifier factor was developed based on a combination of values suggested by Giovinazzi and Lagomarsino (2004) and the proportion of vulnerability reduction for each retrofitting activity suggested by Wang, Sarhosis and Nikitas (2018). It then reports how by using the vulnerability function for existing non-engineered houses developed by IIEES (Ashtiany et al. 2019) and the behaviour modifier factor based on the proportion of score assigned to each retrofitting intervention, a modified vulnerability index and vulnerability function for retrofitted houses were developed in this case study using the model provided by Lagomarsino and Giovinazzi (2006). The results of this study indicate the vulnerability reduction of retrofitted houses.

## Profile of Kabul City

Kabul is the capital and largest city of Afghanistan; its total population is estimated at 3 564 855, of which 41% live in urban areas. There are 962 467 housing units in the city, and the entire city consists of 22 districts (Ministry of Urban Development Affairs 2015).

From April 2017 to March 2019, the PCR implemented various structural and non-structural activities for disaster risk reduction in the two major cities of Kabul and Mazar-i-sharif. One of the structural components of the project was to reinforce 100 houses, of which 50 houses were allocated for Kabul and 50 for Mazar-i-sharif. The targeted areas of the project for this task were limited to six areas in both cities called ‘Gozar Assembly (GA)’ or ‘Gozar’ (three Gozars in Kabul and three in Mazar-i-sharif). A GA is a portion of a district in the city that consists of 1000–1250 houses. Each GA is officially registered under the supervision of the municipality. In Kabul, the registered GAs included GA16 and GA17 in district 13 and GA1 in district 16. However, discussion of the Mazar-i-sharif project is out of the scope of this article. The selection of Gozars for the project was based on a general vulnerability assessment at the city level by the PCR, with suggestions from the municipality (UN-Habitat & PCR 2019).

### Hazard

In 2015, the Ministry of Urban Development of Afghanistan implemented a joint project with the UN-Habitat and other related organisations to survey the status of Afghan cities. The survey found that Afghan cities, including Kabul, are experiencing rapid urbanisation without undertaking adequate disaster risk reduction measures. Because of the concentration of population and industry in urban areas, the residents of Kabul face diverse risks from natural hazards such as earthquakes and floods. Afghanistan is especially vulnerable to earthquakes as the entire country is located on two major active faults that have the potential to rupture and cause extensive damage. Kabul is located in a medium-risk zone of earthquake (Ministry of Urban Development Affairs 2015). However, because of the lack of necessary codes and bylaws for building construction and lack of enforcement of even those that exist, many buildings will not be able to resist even a moderate earthquake (Ministry of Urban Development Affairs 2015). In addition, the poor economic conditions and infrastructure in the city combined with migration from rural areas to urban areas are further accentuating the risks faced by the city (Ministry of Urban Development Affairs 2015).

As mentioned earlier, a recent seismic hazard assessment for Kabul City was undertaken by IIEES as part of PCR activities. The main objective of this task was to clarify and study the existing seismic faults around Kabul City and conduct a detailed seismic hazard analysis at a grid spacing of 1 km. The output of this task was a seismic hazard map for Kabul City for different return periods. The report expresses a high level of seismic hazard in Kabul City in which PGA is close to 0.76 g for 2475 years of return period, which can lead to the occurrence of an earthquake of magnitude of 7.5 (Ashtiany et al. 2019). Notably, the same value was reported to be 0.50 g for the same return period in a previous report by the USGS (Boyd et al. 2007).

### Exposure and overview of non-engineered housing typologies in targeted areas

Related joint reports by the Ministry of Urban Development and Land (MUDL) of Afghanistan with the UN-Habitat declare that ‘Afghanistan has the largest construction material sources, which include aggregate, stone, and brick’. In formal construction projects, cement and concrete account for 23% of the construction material in the country. There are large sources of quarry products that can be used as raw materials for affordable construction and are located near most project sites. Baked and unbaked bricks are also widely being used and account for approximately 16% of construction materials in Afghanistan; they are usually supplied by local production units in the country (Majale 2017). Baked bricks have a compressive strength of 14–21 kg/cm^2^ and they are used for the construction of load-bearing walls. Sun-dried bricks are also popular in construction in Afghanistan and can be distinguished as they easily split into two when tapped against a baked brick (USAID 2012).

According to the ‘State of Afghan Cities’ report by the Ministry of Urban Developments Affairs of Afghanistan, housing development in Afghanistan can be categorised as ‘formal’ and ‘informal’. Formal developments are those that are under the supervision of the government with legal land and legal construction and are as per the master plan, whilst informal developments refer to those that are constructed on illegal land and are not in compliance with the master plan. Certain visible characteristics of informal development include irregular streets, poor quality of construction and lack of services and infrastructure for utilities such as water and electricity. Amongst the 962 467 housing units in Kabul City, 54% constitute informal housing (Ministry of Urban Development Affairs 2015). Bertaud (2005) developed a map of informal settlements for Kabul City, which indicates that 82% of the population in Kabul lives in informal settlements. Compared to engineered buildings, which are properly designed and supervised by engineers and architects, non-engineered buildings are constructed by masons without any supervision or input from engineers. Most such houses are constructed using traditional materials such as sun-dried or burnt bricks, stone and wood. There are certain important factors that affect the level of damage to buildings in the event of a disaster and they are commonly observed in non-engineered construction in Afghanistan. These factors include site conditions, building configuration, large openings in walls, uneven rigidity distribution, lack of ductility, inadequate foundation and poor quality of construction (Arya 2003). During the project period, the author was given the responsibility of developing the procedure for retrofitting the 100 resilient houses in Kabul and Mazar-i-sharif. The first step involved categorising the existing non-engineered houses in both cities. The categorisation was limited to only non-engineered houses targeted by the project, and excluded engineered buildings with steel or concrete frames. The findings of this field survey are described further.

#### Single storey burnt brick masonry with flat ceiling

**FIGURE 3:**
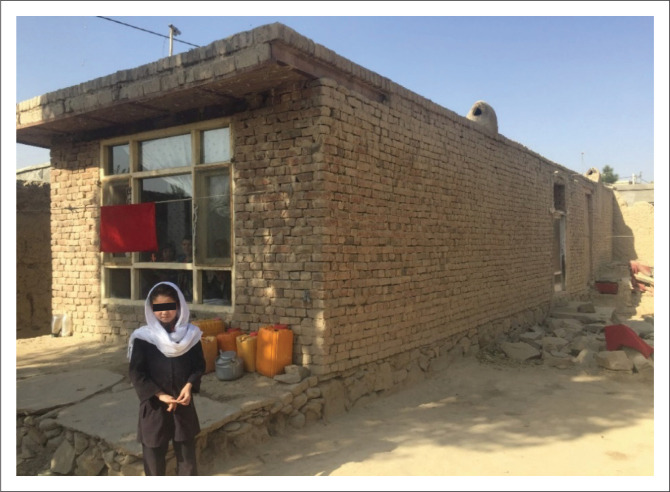
A single-storey burnt brick masonry house with a flat roof.

This type of building is generally constructed with one or two storeys. As shown in Figure 3, the structure consists of load-bearing walls constructed using burnt bricks and cement mortar. The thickness of the load-bearing wall can range from 20 cm to 35 cm. In this typology, the ceiling is covered by placing IPE140 beams at approximately 1 m intervals and filling the distance between them with burnt bricks and gypsum paste. In order to ensure lateral stability of the ceiling, each panel is constructed with a maximum deflection of 2 cm. However, owing to the concave shape of the panels, the plaster thickness from below the ceiling has to be increased in deep parts, which makes the structure heavy because it is a vaulted brick ceiling. In addition, this type of ceiling construction is vulnerable to lateral seismic loads and can easily collapse.

#### Single storey sun-dried clay brick masonry buildings with flat ceilings

**FIGURE 4:**
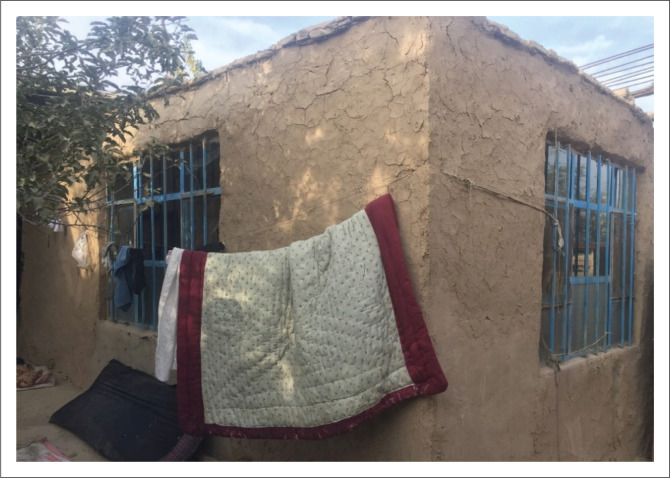
A single-storey mud brick masonry house with a flat roof.

Figure 4 illustrates a sample of sun-dried brick masonry buildings in which the structural system also includes load-bearing walls, but they are constructed using sun-dried clay bricks. In this type of building, the wall thickness is at least 40 cm and, in some cases, it increases to 80 cm. The ceiling is often covered with wooden joists 10 cm – 15 cm in diameter, placed at 50-cm intervals and then covered with a 2 cm – 3 cm thick wooden plate and a 20 cm – 30 cm thick cob for waterproofing. However, this makes the ceiling very heavy. Sometimes, the cob is also used for the wall plaster. In some cases, a basement floor is also constructed in these buildings, which makes them even more vulnerable to lateral seismic loads.

#### Single storey sun-dried brick masonry buildings with barrel- or dome-shaped ceilings

**FIGURE 5:**
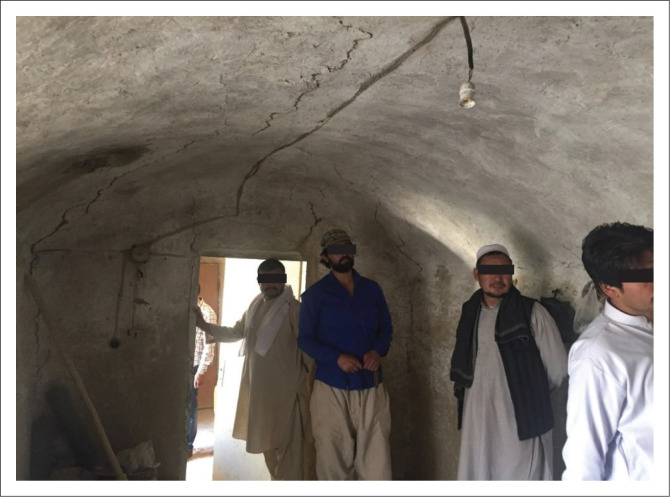
A single-storey mud brick masonry house with a barrel-shaped roof.

This type of building also utilises load-bearing walls with a thickness of 40 cm – 80 cm and are made of sun-dried clay brick. However, the ceiling cover consists of sun-dried bricks and is barrel- or dome-shaped. Figure 5 shows an example with a barrel roof, which has some horizontal and diagonal cracks. These buildings also have weak resistance to lateral loads and can easily collapse.

## Housing selection and preparing proposal

### Preparing modality of selection

As per Output 1.5 of the project, 100 vulnerable houses in the two target cities of PCR had to be selected for retrofitting. The targeted area for Kabul City included three GAs in districts 13 and 16. Regarding this activity, the PCR developed a modality for the selection of vulnerable houses (see Online Appendix 1). The modality mainly consisted of two major parts: one part for listing the social criteria (score of 30 out of 100) and another part allocated for technical criteria (70 out of 100). The former generally evaluated the economic condition of each family based on the number of workers, dependents, average monthly income and number of old and disabled members in each family. The technical part encompassed criteria associated with the engineering-based evaluation of a house. These criteria mainly included the location of the house, foundation, roof condition, cracks on walls, quality of materials and joint connections. This document was also shared with the municipalities for their review and input. In the meetings held with municipalities, the concept, types and criteria score, as well as the overall procedure of selection were shared. The municipalities were requested to nominate a technical person as a point of contact for participating in the technical training of the modality of selection of vulnerable houses and to supervise the actual on-site housing assessment activities. Furthermore, the list of selected houses for retrofitting was shared with the municipalities for their approval. Based on the modality of selection, each of the three GAs in Kabul nominated 50 houses and a total of 150 houses were listed. The technical team from the project used social and technical criteria to assign a score to each house. In the end, 18 houses with the highest score in each GA were short listed and, in total, there were 54 houses in Kabul. However, because of some social issues, 6 houses were cancelled at the last moment and 48 houses were selected for retrofitting.

### Training for vulnerable housing assessment

To apply the procedure of selection of houses on site, the prepared modality of selection of vulnerable houses was presented to the PCR team in the presence of nominated persons from the municipality. In the training, the social and technical criteria, scoring and method of capturing each criterion were explained to the participants.

### Explaining the procedure of housing selection for GA

At the GA meeting, the concept of retrofitting and procedure of housing selection were explained to GA members and they were familiarised with the objective of the retrofitting activities. As mentioned earlier, each GA was requested to make a list of 50 houses that were structurally weak and vulnerable and inhabited by the poorest people from the community. In addition, it was stated that for each GA, around 15–17 houses will be selected for retrofitting from the 50 nominated ones. Because of future probable social challenges amongst people, site engineers were instructed to explain the concept and procedure of selection for house owners once more on the day of the house visit.

### Vulnerable housing visit and assessment

The assessment of vulnerable houses was conducted under the supervision of representatives from the municipality. The social questionnaire relating to the number of family members, condition of household head, extremely vulnerable individuals in the family and level of income was completed by social organisers for each house. Simultaneously, the site engineer took responsibility for the technical assessment of houses, which mainly consisted of site and soil conditions, architectural conditions, material and structural conditions (Online Appendix 1).

After assessing the houses that had been nominated from each GA, those houses that had the highest score in each GA were targeted for retrofitting. As mentioned earlier, 104 houses were finalised in this manner, of which 54 (later, 48) belonged to Kabul and 50 to Mazar-i-sharif.

### Preparing proposal and training of local masons

Immediately after finalising the targeted houses for retrofitting, the field team was instructed to prepare a sketch of each house and take photographs. In addition, a relocation plan that included temporary tents for displacement of each household during actual retrofitting activities was considered by the project and explained to each household. Design drawings for retrofitting of houses were undertaken and supported by UN-Habitat’s Iran office, and a retrofitting design was developed based on the level of life safety. Retrofitting design at this level which is normally considered for residential buildings will give residents enough time to evacuate and save their life when an earthquake occurs. Calculation of quantities and preparation of the proposal were conducted in two batches for each GA.

### Training of local masons

The PCR held technical training sessions for the local masons and welders on resilient housing construction and retrofitting with the cooperation of technical experts from UN-Habitat’s Iran office. The training consisted of theoretical and practical sessions. In the theory sessions, a general understanding of natural hazards such as earthquakes, failure mechanisms of houses, common construction problems and safe construction and retrofitting principles were taught and discussed. After the theory session, the participants were taken to the site of one of the targeted houses for retrofitting and all retrofitting activities were implemented under the supervision of the trainers.

## Seismic retrofitting

The retrofitting work performed on the houses is described in the following sub-sections.

### Additional reinforced cement concrete foundation ring

**FIGURE 6:**
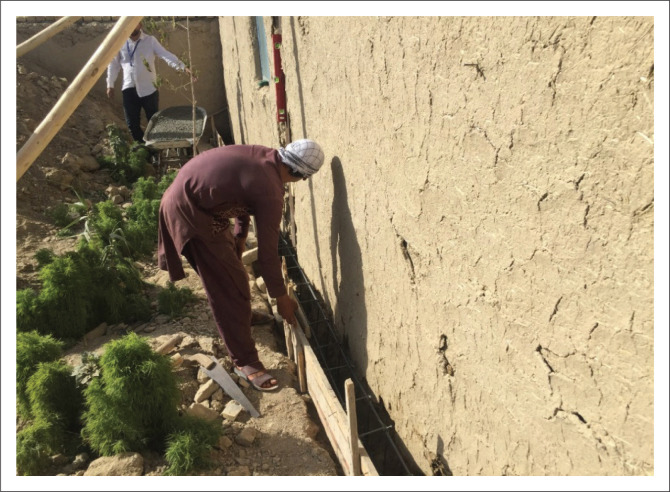
Implementing an additional RCC foundation ring.

For masonry houses without any foundation, an RCC ring (60 cm × 50 cm beams) was tied around the structure near the bottom on the outer side of the walls to ensure the house’s stability during an earthquake, as shown in Figure 6. For this task, masons excavated and levelled the area around the house, constructed a 1 cm – 2 cm layer of plain cement concrete (PCC), added steel bars reinforcements, performed shuttering and then prepared concrete. The excavation of the foundation ring was carried out carefully until an appropriate levelled surface was achieved. In special cases, to ensure stability of the existing walls, additional wooden or steel supports were considered.

The 28-day cylinder strength of the concrete for this element was 250 kg/cm^2^. The mix design included 2 parts gravel, 2 parts sand, 0.5 part cement and 1 part water. During the actual work, for one bag of cement, at least 30 L of water was considered. The water used for the concrete was clean, without any additional materials. After finishing the concrete work, it was kept wet for 14 days post implementation.

The reinforcement used for this purpose contained six D10 ribbed steel bars at the top and bottom with D8 stirrups at each 25 cm interval. In addition to horizontal bars and stirrups, the RCC ring needed to be attached to the existing wall. For this purpose, three L-shaped D10 additional bars (30 cm length) at 25 cm intervals were applied. The steel bars used for the concrete works were grade 60, with a tensile strength of 400 MPa.

In certain cases, the thickness of the stone masonry under the existing wall was greater than the wall thickness (with approximately 10 cm – 20 cm ledge); in such cases, the foundation ring was installed in addition to existing stone masonry.

### Horizontal and vertical ties

The common construction culture for masonry buildings in Afghanistan mainly includes the construction of load-bearing masonry walls with 30 cm – 40 cm thick sun-dried bricks and roof cover with wooden or steel joists, plywood cover and 15 cm – 20 cm mud mortar as the final cover. The weak connection between the roof and the walls and between the adjacent walls in such buildings leads to an incomplete load path at the time of occurrence of an earthquake.

**FIGURE 7:**
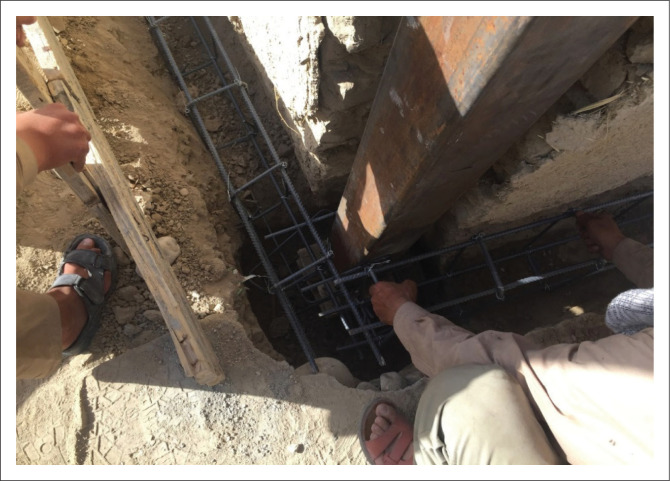
Placing vertical tie inside the foundation ring.

Providing horizontal and vertical ties for non-engineered buildings was another retrofitting task that made the buildings much more integrated and resistant to lateral loads. For this purpose, the application of boxed 140 mm × 140 mm vertical ties and UNP160 horizontal ties was suggested. In most cases, vertical ties were placed inside the wall thickness from outside the building, and they were connected to the RCC foundation ring, as shown in Figure 7. However, there were certain cases where it was not possible to place the vertical tie outside the building; for example, when the wall was adjacent to the neighbour’s wall, in such cases, the vertical tie was implemented inside the building with proper connection to the existing wall.

In houses with the roof cover in a poor condition, the roof was demolished and a horizontal ring was installed in the wall thickness. In the connection points of the horizontal and vertical ties, an L100 × 100 × 10 mm angle with a 90 × 90 × 10 mm plate inside it was welded with D = 4 mm. The horizontal ties were fixed on the wall thickness via U-shaped D8 steel bars at 50 cm intervals to ensure good connection between the horizontal tie and the existing masonry wall. In cases where the existing roof was in good condition, horizontal ties were installed inside the house under the existing roof. In such cases, an appropriate connection between existing joists and new horizontal ties was considered. In addition, vertical ties adhered to the existing wall with a D8 steel strap at 75 cm intervals. These straps were threaded on both sides and tightened through nuts and bolts with a steel plate on the other side of the wall. In cases where access to the other side of the wall was impossible (i.e. because of a neighbouring house), the straps were inserted inside the wall and any empty space was filled with grout. In order to make an appropriate connection and to complete the load path between the vertical tie and the RCC foundation, additional steel bars were welded at the bottom of the vertical tie where it was connected to the foundation.

As mentioned earlier, there were some sun-dried brick houses with barrel- or dome-shaped roofs as well. However, such roofs were demolished as they are very heavy and, subsequently, have weak resistance to lateral loads. In most such houses, the thickness of the load-bearing walls was at least 60 cm. In such cases, horizontal ties with RCC on wall thickness were implemented.

**FIGURE 8:**
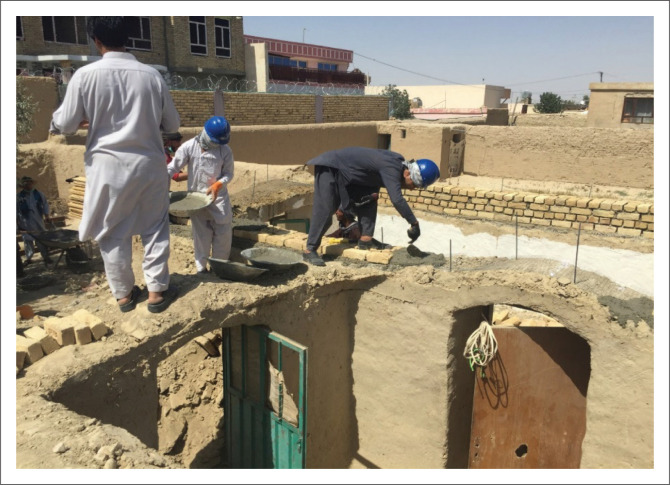
Additional brick works on the top of the existing wall and making connection using steel bars.

After demolishing the roof and before applying the horizontal ties, some additional brick works on the top of the existing wall were performed, and a steel bar and a mesh were used to properly connect the RCC ring to the existing wall, as shown in Figure 8. To ensure good connection, additional 2 m long D12 steel bars were applied at 50 cm intervals. The lengths of these steel bars were reduced for the top of the openings. A drilling machine was used to measure the wall thickness before implementing these straps. The horizontal tie for this purpose was 30 cm in height and its width was equal to the wall thickness. There were four D10 horizontal steel bars, and D8 stirrups were placed at 25 cm intervals.

### New ceiling cover

**FIGURE 9:**
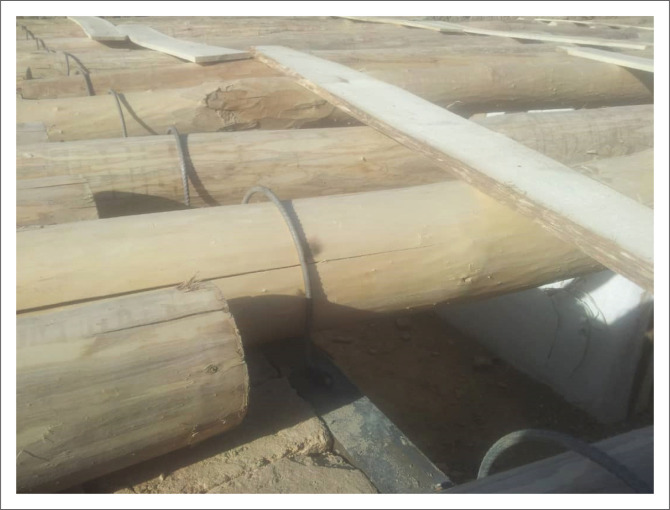
New roof cover and its connection with horizontal ties.

New steel or wooden joists used for ceiling cover were placed at 50 cm intervals and connected to the horizontal ring. The joists used for the project were wooden; however, in some cases, steel profile ST37 IPE or UPE140 was applied. The connection between the new steel joist and steel horizontal tie was welded together through a L100 × 100 × 10 angle. In cases where the horizontal tie was RCC, new steel joists were placed inside the RCC horizontal tie reinforcement before concrete in order to provide a connection between these elements. In addition, the distance between the stirrups around the connection point of the steel joist to the RCC horizontal tie was reduced to 10 cm at a length of 50 cm. Wooden joists used for the ceiling cover were 15 cm in diameter, straight and without any visible cracks. Good connection between the wooden joist and the steel horizontal tie, as shown in Figure 9, was ensured through the use of U-shaped D8 steel bars placed on the wooden joists and welded to the horizontal tie. To prevent termites, both sides of the wooden joists were covered with a layer of bitumen before installation.

X bracing with a D12 steel bar on the top of wooden joists was implemented to ensure its resistance and integrity to lateral loads. These steel bar bracings were tightened at both ends of the frame. Implementing a small trench on the wooden joist where the X bracing steel bar passes make the wooden plate installation easy. The steel bar was fastened to a wooden joist using dowels. A new wooden plate (2.5 cm thick) was used on top of the wooden joists together with a plastic sheet and 8 cm RCC on top of it. To prevent moisture in the ceiling, an isogam cover on the surface of the concrete roof was applied after 14 days.

### Wire mesh and plaster

In most masonry houses in the project-targeted areas, load-bearing walls constructed with sun-dried brick and mud mortar did not possess sufficient shearing capacity against lateral loads, and this issue contributed to increasing the seismic vulnerability of such houses.

**FIGURE 10:**
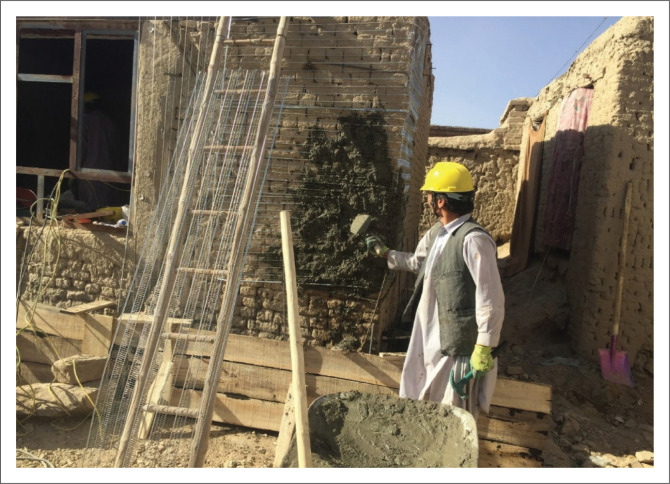
Wall mesh and plaster.

To overcome this deficiency, a layer of steel wire mesh (1 mm – 2 mm diameter) and plaster was used on the wall surface (as indicated in Figure 10). Firstly, it was necessary to remove the existing plaster on the wall. In addition, small 1 m trenches at 45° were made in the corners of the openings. Inside each trench, two 1 m long D8 steel bars were placed and fixed to the wall with dowels. Similarly, 2 m long trenches (1 m at each side) on the outside corner of the walls were made at 60 cm intervals. Inside each of these trenches, two 2 m long D8 steel bars (1 m on each side) were inserted and fixed to the wall with dowels. The surface of the wall was wettened with water in order to prevent water absorption from the plaster. The wire mesh was installed on the wall surface using L-shaped dowels made from 30 cm long D6 steel bars at 50 cm intervals. Finally, a 3-cm thick cement plaster (consisting of one part cement, four parts sand and water) was applied on the wall surface.

### Strengthening masonry houses with basement

**FIGURE 11:**
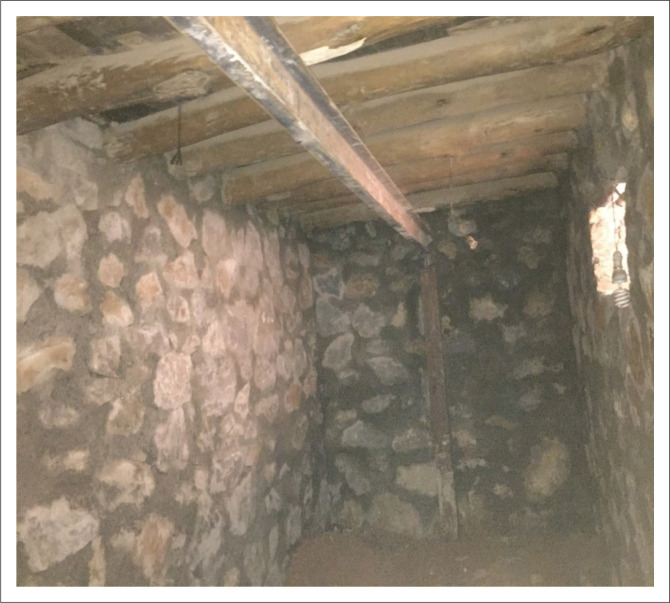
Additional stone masonry for basement improvement.

Generally, in masonry houses, it is not possible to construct a basement as they make the structure vulnerable to disasters. To retrofit existing masonry houses with a basement, necessary actions to strengthen the walls in the basement were considered, and additional supporting columns with a beam under the ceiling of the basement were installed. Because there was a storey above the basement, a stone masonry retaining wall with cement mortar was added as support and buttress of the basement wall, as shown in Figure 11. Additionally, boxed 140 × 140 columns were also used for this purpose and the beam profile was UNP160 and IPE160.

### Strengthening masonry houses with two storeys

The general design considered for two-storey buildings was similar to that for one-storey houses. However, we considered a steel bracing system in addition to a primary steel frame to ensure compliance with an adequate resisting system. The cross-section of the bracing was L50 × 50 × 5 two spans for each direction. However, steel bracing was implemented only for buildings that had steel frames. In the case of an existing ceiling built using wooden joists, it was replaced with a suitable joist and light insulation. In the implementation phase, the ceiling of the second floor could easily be replaced with a new one; however, necessary safety measures to ensure the stability of the entire building was considered for the replacement of the ceiling in the first floor. An additional steel frame was installed adjacent to the existing wall, and the existing mud mortar and wooden plate on the ceiling of the first floor were removed carefully. New steel joists (UPE140 or IPE140) at 1–1.5 m intervals were installed and welded to the steel frame.

In the case of this project, because the steel frame was outside the building, dismantling a small part of the wall to pass the steel joist was carried out. After installing new steel joists, the existing wooden joists were removed with a saw. Subsequently, further tasks for installing a new wooden plate (with 3 cm thickness), reinforcement and 8 cm concrete were carried out.

### New buttress

**FIGURE 12:**
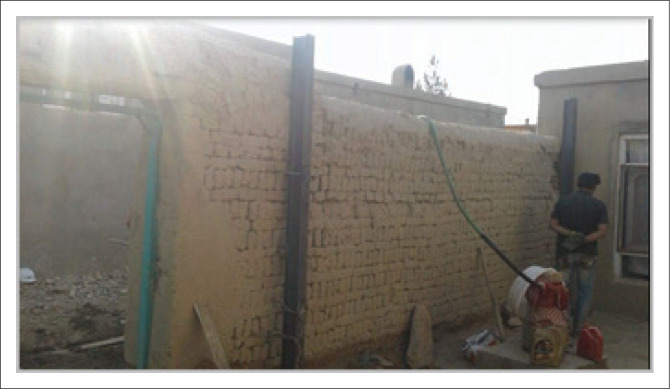
Installing buttress for surrounding wall.

Boundary walls, such as yard walls in masonry buildings, are more exposed to the risk of overturning because of floods or earthquakes. To increase the resistance of such walls, installation of additional vertical ties behind the wall (as indicated in Figure 12) was considered. These IPE140 ties were installed at 5 m intervals behind the existing boundary walls and in an 50 cm × 50 cm RCC foundation located at a depth of 40 cm. For a good connection between the steel column and concrete in the foundation, additional steel bars were welded at the bottom of the column. In addition, the vertical ties were adhered to the wall using steel straps at 50 cm intervals.

To ensure the overall stability of the wall, two diagonal D8 or D10 bars between the columns (vertical ties) and a horizontal D8 or D10 bar placed near the top of the wall were attached at 1 m intervals using straps. This included: creating T-sections with the bars on the earth, making holes in the wall thickness across the diagonals and horizontal bars, bending the bar on the main diagonal or horizontal bar and providing a connection with them using small welds or proper steel ties.

## Methodology

This study used a combination of reducing vulnerability for each retrofitting intervention suggested by Wang et al. (2018) and retrofitting intervention as components of the behaviour modifier factor for the vulnerability index suggested by Giovinazzi and Lagomarsino (2004). The term *vulnerability* explains the extent of damage associated with an element at risk (i.e. a building) at a specific level of hazard (i.e. an earthquake), and it is expressed as a ratio between expected loss and maximum possible loss (Coburn & Spence 2002). In order to evaluate the level of vulnerability of an existing building, it is possible to rely on the estimated level of performance based on calculation and expert judgement, which is called *predicted vulnerability*. This method is more suitable for structures in which a reasonable estimate of earthquake resistance can be made. It is also possible to estimate vulnerability using previous data on earthquake damage where the data are available. This method is called *observed vulnerability* (Coburn & Spence 2002).

Vulnerability is determined based on a vulnerability index V and a ductility index Q, both of which are estimated in accordance with the building typology and construction material (Coburn & Spence 2002). The extent of physical damage based on European Macroseismic Scale issued in 1998 (EMS-98) is classified by a parameter called *damage grade*, which is expressed based on a scale from D1 (slight damage) to D5 (complete destruction) for a building element (Coburn & Spence 2002). In order to state this parameter for a specific number of buildings, another parameter called *mean damage grade* is used (Coburn & Spence 2002). Using a vulnerability index, which is the main parameter for deriving the vulnerability function of a specific type of structure, the mean damage grade can be calculated as follows (Lagomarsino & Giovinazzi 2006):

$$\mu_{D}=2.5\left[1+\tanh\left(\frac{I+6.25.V_{I}-13.1}{2.3}\right)\right]$$ [Eqn 1]

In Equation 1, *μ_D_* is the mean damage grade, parameter *I* is the intensity of the earthquake and *V_I_* is the vulnerability index.

By *μ_D_*, the damage ratio *D_i_* can be calculated using Equation 2 (Lagomarsino & Giovinazzi 2006):

$$D_{i}=-0.0004\mu_{D}^{3}+0.0854\mu_{D}^{2}+0.0085\mu_{D}$$ [Eqn 2]

The vulnerability index depends on structural elements, building materials, site situations and other interventions. Equation 3 provides a definition by the European Macroseismic Scale (EMS-98) (Lagomarsino & Giovinazzi 2006):

$$\bar{V_{I}}=V_{I}^{*}+\Delta V_{R}+\Delta V_{m}$$ [Eqn 3]

In Equation 3, V_I_^*^ refers to the typological vulnerability index, Δ*V_R_* is the regional vulnerability factor and Δ*V_m_* represents the behaviour modifier factor. The regional vulnerability factor is defined based on specific construction materials and techniques of a region, and expert judgement specifies the extent to which it modifies the typological vulnerability index. The behaviour modifier factor is evaluated as a summation of scores given to various structural factors such as structural systems, plans, roofs, floors and retrofitting interventions (Giovinazzi & Lagomarsino 2004).

Other related studies have also evaluated the application of EMS-98 for the assessment of buildings in Asia and the Pacific regions, including Pakistan, which has similar building taxonomies with minor differences compared to Afghanistan. The study also declares a high level of vulnerability associated with the majority of buildings that are unreinforced masonry buildings in moderate-to-severe earthquake strikes. Furthermore, it states the easy applicability of EMS-98 outside Europe with a robust methodology for damage, vulnerability and macroseismic intensity evaluation (Maqsood, Schwarz & Edwards 2013).

**FIGURE 13:**
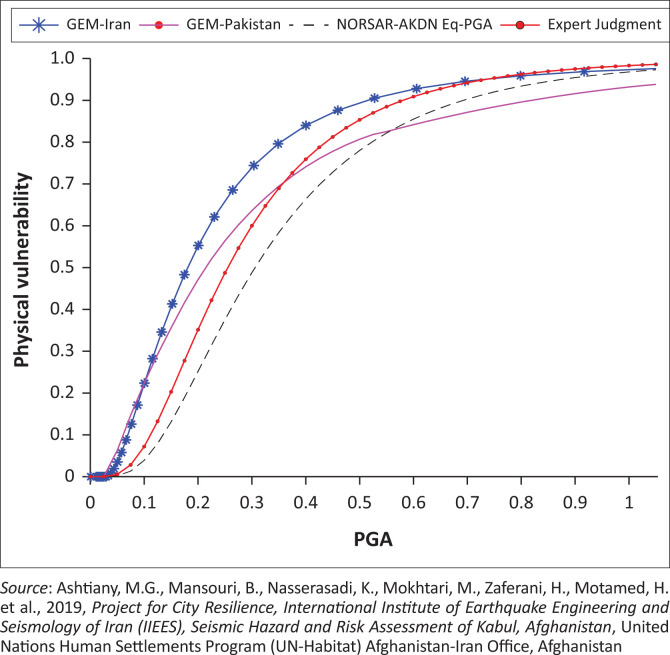
Comparison of developed vulnerability function for adobe structures.

The vulnerability functions for buildings in Afghanistan were developed by the International Institute of Earthquake Engineering and Seismology (IIEES) of Iran (Ashtiany et al. 2019) as a contract work package for UN-Habitat’s Afghanistan office in 2019. Figure 13 shows the vulnerability curve for adobe buildings, which were derived via numerical analysis and site visits of mud brick adobe houses in districts 13 and 16 of Kabul City, and it has been compared with similar previous related works conducted by the Global Earthquake Model (GEM) Iran, GEM Pakistan and Norsar Akha Khan foundation. According to the developed vulnerability curves, existing adobe buildings in Kabul City will experience 60% damage at 0.3 g PGA and more than 90% damage at a PGA of 0.6 g and higher. The vulnerability function in Figure 13 was developed with a vulnerability index of 0.817 for sun-dried clay brick adobe buildings in Afghanistan. It is also remarkable that vulnerability functions for building typologies in Afghanistan have been developed using central damage factor values based on the HAZUS methodology with the incorporation of values suggested by EMS-98 (Ashtiany et al. 2019).

**FIGURE 14:**
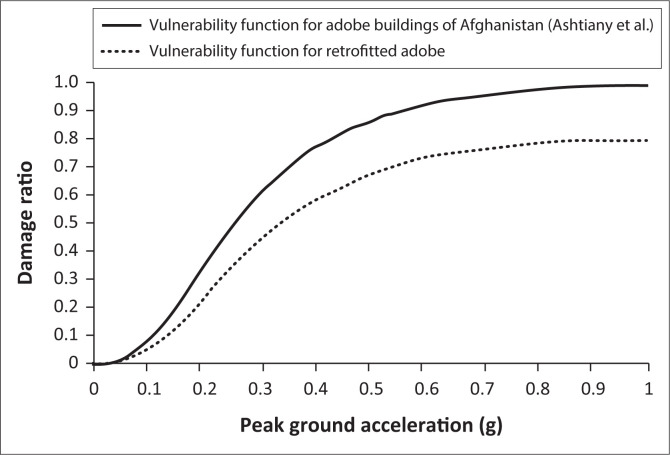
Effect of retrofitting on vulnerability reduction.

To estimate the vulnerability index for retrofitted sun-dried clay brick adobe buildings, the major retrofitting activities in 48 retrofitted buildings by PCR were assessed. The data for each house were obtained from the PCR database and the author’s field survey. The field survey was conducted house by house after completion of retrofitting activities, and each task was evaluated on-site based on actual work and the proposed design. Activities such as installation of an additional foundation, additional frame, new ceiling cover and wall strengthening (mesh and plaster) were considered as behaviour modifier factors in retrofitting intervention, which could reduce the vulnerability index according to the values suggested by Giovinazzi and Lagomarsino (2004). Each retrofitting activity conducted in this case study had a specific effect on increasing the resistance of the building and reducing seismic vulnerability. Related reports on past earthquakes that occurred in some parts of Afghanistan and Pakistan have recorded abundant damage to non-engineered unreinforced masonry and adobe buildings, which were mostly because of out-of-plane collapse of the boundary wall and load-bearing walls and partial collapse of the roof (Ismail, Mipenz & Khattak 2015). In another related study by Wang et al. (2018), existing retrofitting methods, together with their advantages and shortcomings, have been reviewed. It states that retrofitting methods have different impacts on increasing the strength of the building. However, there is no best method, and each method needs to be decided by the engineer on-site depending on the characteristics of the house and its materials. The study summarises a comparison of various retrofitting methods. It indicates that by using the wall mesh and plaster, the resistance of the existing wall will reach 150%. The reticulatus system can increase the resistance of existing stone masonry by approximately 40%, and by external steel reinforcement, the lateral in-plane resistance is improved by a factor of 4.5 (Wang et al. 2018). The allocation of scores to our retrofitting activities was decided based on the proportion of its effect on increasing the strength of the building, as suggested by Wang et al., whether it is implemented completely and without defects. Table 1 shows the scores of the implemented retrofitting intervention as a behaviour modifier factor, which is suggested to be - 0.08 by Giovinazzi and Lagomarsino (2004). This score was distributed amongst three main retrofitting tasks ‒ additional foundation, additional frame with ceiling replacement and wall strengthening (wall mesh and plaster) ‒ according to the extent of seismic improvement for each activity (Wang et al. 2018) and it was applied for each of the 48 houses in the author’s field survey after completion of the retrofitting task. However, based on the author’s supervision on site, in case of defects in implementation, the score was adjusted according to the structural analysis of the implemented retrofitting task. After assessment of 48 houses by allocating retrofitting intervention scores for each house, the mean score for each retrofitting activity across all houses was calculated, and the overall score of retrofitting intervention for all retrofitted houses was estimated as shown in Table 2. Using Equation 3, the estimated value for the behaviour modifier factor was subtracted from the original vulnerability index for sun-dried brick adobe houses in the original vulnerability function developed by Ashtiany et al. (2019). It is also noteworthy that the effect of the regional vulnerability factor has already been considered in the estimation of vulnerability function for adobe buildings in Afghanistan by Ashtiany et al. (2019). The estimated vulnerability index for retrofitted buildings to develop a vulnerability curve was used from PGA = 0 to PGA = 1 at intervals of 0.025 (the same as considered by Ashtiany et al. 2019). The PGA in each interval was then converted to intensity using a mid-curve which is the result of different relationship between intensity and PGA in the literatures (Omidvar, Gatmiri & Derakhshan 2012). The intensity and modified vulnerability index were used in Equation 1 to calculate the mean damage grade, which was then used in Equation 2 to calculate the damage ratio from PGA = 0 to PGA = 1 at each 0.025 interval. Figure 14 shows both vulnerability curves for retrofitted sun-dried clay brick adobe buildings and the vulnerability curve developed by Ashtiany et al. (2019).

**TABLE 1:** Allocation of scores to retrofitting intervention.

Retrofitting intervention	Behaviour modifier factor
Additional foundation	−0.0151
Additional frame with roof replacement	−0.0486
Wall mesh and plaster	−0.0162

*Source*: Adapted from Giovinazzi and Lagomarsino 2004; Wang et al. 2018:251–268.

**TABLE 2:** Developing the behaviour modifier factor for implemented retrofitting task.

Retrofitting intervention	Behaviour modifier factor
Additional foundation	−0.0132
Additional frame with roof replacement	−0.0294
Wall mesh and plaster	−0.0140
**Total**	**−0.0566**

### Ethical considerations

This article followed all ethical standards for conducting the research.

## Results and discussion

**FIGURE 15:**
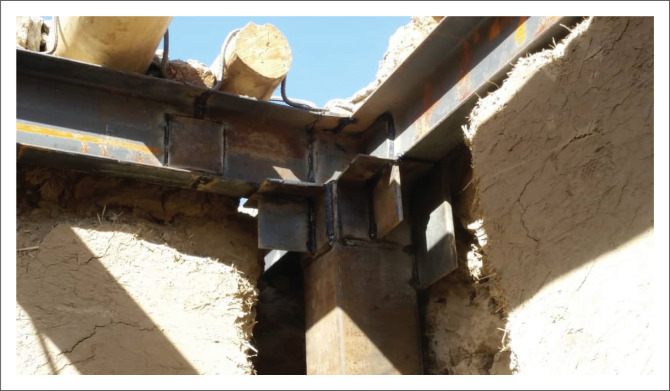
Splice in beam.

**FIGURE 16:**
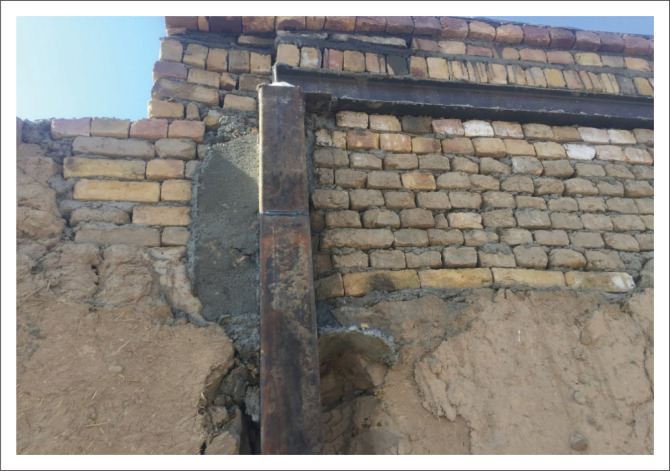
Splice in column.

As mentioned above, retrofitting interventions were given a proportion of the score suggested by Wang et al. (2018) as a behaviour modifier factor using values suggested by Giovinazzi and Lagomarsino (2004) as shown in Table 1. Table 2 shows the score based on actual work done by local masons and welders. According to Table 2, the highest proportion of seismic vulnerability reduction in 48 retrofitted houses belongs to ‘additional frame with ceiling replacement’, with a score of -0.0294. The other two retrofitting interventions, ‘wall strengthening (mesh and plaster)’ and ‘additional foundation’ with scores of -0.0140 and -0.0132, respectively, seem to have a relatively lower effect on vulnerability reduction compared to ‘additional frame with ceiling replacement’. It can be seen from Table 3 that the local masons and welders obtained 60.42% for ‘additional frame and ceiling replacement’. Although this score is less than the other two interventions, it still has the highest share in vulnerability reduction in this retrofitting practice. However, this issue indicates that the quality of the welding task and steel frame construction work needs to be improved in the future. Figure 15 and 16 show the additional frame and welding task. In some houses, to save steel material and costs, some beams and columns were assembled with splices that were located near the beam or column end, where the shear force is greater than the other part of the frame. Because of the novelty of such retrofitting of sun-dried brick dwellings amongst local communities of Afghanistan, some defects in implementation were anticipated. However, these defects do not significantly influence the entire retrofitting quality because retrofitting will not occur if it reduces the strength of the existing building. In general, these are still reasonably good scores for local masons and welders who did not have a solid base of previous experience in seismic-resistant construction. In other words, through PCR’s contribution to enhance the capacity of local community via technical training on resilient housing construction and retrofitting, they were able to carry out such retrofitting intervention in Kabul City.

**TABLE 3:** Average score achieved by local masons and welders.

Retrofitting intervention	Score (%)
Additional foundation	87.58
Additional frame and roof replacement	60.42
Wall mesh and plaster	86.46

The overall score for retrofitting intervention in the PCR for the case of Kabul City was estimated at -0.0566. This score, which represents the behaviour modifier factor in retrofitted buildings, changes the vulnerability index from 0.817 in the original vulnerability function of adobe buildings by Ashtiany et al. (2019) to 0.761. Accordingly, a vulnerability curve for retrofitted buildings has been developed. Figure 14 shows a comparison of the vulnerability curves for sun-dried clay brick adobe buildings before and after retrofitting. According to the figure, the damage ratio of adobe buildings has been reduced by 15%, at PGA 0.3 g and maximum 20% for higher ground motion. This level of damage reduction will give residents enough time to evacuate and save their lives in the event of an earthquake.

In another related study by Arya (2000), non-engineered construction in developing countries was discussed. This study classified building typologies, including adobes with unburnt brick, ordinary brick buildings with half-timber and reinforced concrete buildings. The retrofitting method for masonry houses presented within the study was reinforced concrete seismic bands with connections in the corners for all external and internal walls at different levels, including lintel as a horizontal element and using steel bars or bamboo as vertical elements. In addition, a similar approach using bamboo for horizontal and vertical seismic resistant elements was suggested for strengthening adobe earthen houses. The vulnerability functions, developed in the study, encompass the mentioned building typologies including earthen adobe and masonry in original form and with earthquake-resistant measures, and they have been developed based on the mentioned retrofitting methods. Based on findings of the study for adobe houses (A-type), the average loss ratio shows an approximately 15% decrease at PGA = 0.1 g. When PGA increases to 0.2 g and 0.3 g, the graph shows a greater reduction in the average loss ratio, which is approximately 20% and 22%, respectively (Arya 2000). Our results for the case study of Kabul, however, show less reduction in the damage ratio. Based on Figure 14, after retrofitting sun-dried clay brick houses, the damage ratio decreased by approximately 3% at PGA = 0.1 g, and for higher PGA values of 0.2 g and 0.3 g, the vulnerability reduction was 11% and 15%, respectively. However, for higher ground motion at PGA = 0.6 g, the vulnerability reduction for both cases of the study is approximately 22%.

## Conclusion

The increasing construction of non-engineered buildings in Afghanistan because of its poor economic situation and lack of knowledge associated with seismic resistant construction has exposed a large percentage of the local community, especially in Kabul City, to a higher risk of damage from future earthquakes.

This study presented a retrofitting practice and methods that were implemented by the PCR in Kabul City, conducted by UN-Habitat from April 2017 to March 2019 in cooperation with the Government of Afghanistan. The major retrofitting tasks, including additional foundation, additional frame with ceiling replacement and wall strengthening, were assessed after completion of retrofitting activities to develop a vulnerability index and vulnerability function for retrofitted buildings. For this purpose, equations and values suggested by Giovinazzi and Lagomarsino (2004) were used and incorporated with the proportion of vulnerability reduction for each retrofitting activity suggested by Wang et al. (2018). Based on the results, which were compared with similar studies, the retrofitting intervention applied in this case study could help reduce the vulnerability of existing non-engineered buildings, and local masons and welders assigned for this task achieved reasonable scores. Finally, the methods mentioned in this study can be used to make existing sun-dried clay brick masonry buildings sufficiently resistant to earthquakes. It is also suggested that issues regarding seismic resistant construction and retrofitting be incorporated into Afghanistan’s national building codes in an easy-to-understand manner to ensure implementation on ground.
